# Alphastatin-C a new inhibitor of endothelial cell activation is a pro-arteriogenic agent *in vivo* and retards B16-F10 melanoma growth in a preclinical model

**DOI:** 10.18632/oncotarget.27839

**Published:** 2020-12-22

**Authors:** Adilson Kleber Ferreira, Brunella Cristofaro, Milene Cristina Menezes, Ana Karina de Oliveira, Alexandre Keiji Tashima, Robson Lopes de Melo, Cristiane Castilho Fernandes Silva, Miryam Guillermina Palomino Rodriguez, Daniela Cajado de Oliveira Souza Carvalho, Ricardo Alexandre de Azevedo, Paulo Luiz de Sá Junior, Lisley Inata Mambelli, Fernanda Vieira Portaro, Luc Pardanaud, Anne Eichmann, Osvaldo Augusto Sant’Anna, Mxarcella Faria

**Affiliations:** ^1^ Special Laboratory of Applied Toxinology, Center of Toxins, Immune-Response and Cell Signaling (CeTICS), Butantan Institute, São Paulo, SP, Brazil; ^2^ Department of Immunology, Laboratory of Tumor Immunology, Institute of Biomedical Science, University of Sao Paulo, Sao Paulo, SP, Brazil; ^3^ Alchemypet, Veterinary Dignostic Medicine, CIETEC/IPEN, Department of Oncology, University of Sao Paulo, Sao Paulo, Brazil; ^4^ Immunochemistry Laboratory, Butantan Institute, São Paulo, SP, Brazil; ^5^ Cardiovascular Research Center and the Department of Cellular and Molecular Physiology, Yale University School of Medicine, New Haven, CT, USA; ^6^ INSERM U970, Paris Cardiovascular Research Center, Paris, France; ^7^ Mogi das Cruzes University (UMC), Villa Lobos Campus, Sao Paulo, SP, Brazil; ^8^ Center for Interdisciplinary Research in Biology (CIRB), Collège de France, Paris, France; ^9^ Department of Biochemistry, Escola Paulista de Medicina, Federal University of Sao Paulo, Sao Paulo, Brazil; ^10^ Experimental Oncology Unit (UNONEX), Federal University of Sao Paulo, Sao Paulo, SP, Brazil

**Keywords:** alphastatin-C, angiogenesis, peptides, melanoma, angiostatic

## Abstract

Most characterized angiogenic modulators are proteolytic fragments of structural plasma and/or matrix components. Herein, we have identified a novel anti-angiogenic peptide generated by the *in vitro* hydrolysis of the C-terminal moiety of the fibrinogen alpha chain, produced by the snake venom metalloprotease bothropasin (SVMP), a hemorrhagic proteinase in Bothrops jararaca venom. The 14-amino acids peptide (alphastatin-C) is a potent antagonist of basic fibroblast growth factor, induced endothelial cell (HUVEC-CS) proliferation, migration and capillary tube formation in matrigel. It also inhibits cell adhesion to fibronectin. The basis of the antagonism between bFGF and alphastatin-C is elucidated by the inhibition of various bFGF induced signaling pathways and their molecular components modification, whenever the combination of the stimuli is provided, in comparison to the treatment with bFGF only. To corroborate to the potential therapeutic use of alphastatin-C, we have chosen to perform *in vivo* assays in two distinct angiogenic settings. In chick model, alphastatin-C inhibits chorioallantoic membrane angiogenesis. In mouse, it efficiently reduces tumor number and volume in a melanoma model, due to the impairment of tumor neovascularization in treated mice. In contrast, we show that the alphastatin-C peptide induces arteriogenesis, increasing pial collateral density in neonate mice. alphastatin-C is an efficient new antiangiogenic FGF-associated agent *in vitro*, it is an inhibitor of embryonic and tumor vascularization *in vivo* while, it is an arteriogenic agent. The results also suggest that SVMPs can be used as *in vitro* biochemical tools to process plasma and/or matrix macromolecular components unraveling new angiostatic peptides.

## INTRODUCTION

The mammalian circulatory system must adapt to changing systemic and environmental conditions, assuring that the plasma and the matrix composition surrounding the endothelial cells layering the vascular beds adjust in response to major constraints such as changes in oxygen availability and shear stress status. Integrating these multiple inputs, mature endothelial cells (ECs) can modulate their phenotype to activate an angiogenic program, both during normal physiological changes (e.g. vascularization of the endometrium during menstrual cycle) and pathological conditions (e.g. inflammation and tumorigenesis) that would require the formation of new blood vessels from a preexistent network [1]. Under pathological circumstances, a characteristic first step for angiogenesis is the increase of endothelial cell permeability, allowing the extravasation of plasma components and inflammatory cells to the subendothelial space and the deposition of a fibrin-rich provisional matrix [2]. These local changes end up activating endothelial cells and triggering an angiogenic response, which involves the coordination of migration, proliferation and differential adhesion of subsets of endothelial cell populations [3–5]. The resulting vascularization thus initiated can then progress into normal tissue repair, with vascular regression and the restoration of homeostasis, or it can develop into acute inflammation. Ultimately the development of vascularization into tissue repair or chronic inflammation depends on the composition and the turnover of the extracellular matrix (ECM) to stimulate endothelial cell receptors.

The whole multistep process of angiogenesis is controlled by a finely tuned balance between positive and negative effectors in the so-called angiogenic cocktail. An important class of such effectors is the matrikines, cryptic proteolytic products of otherwise structural extracellular matrix components [6]. The continuously growing list of matrikines includes fragments from various macromolecular substrates, such as collagens, perlecan, thrombospondin, fibronectin, laminin [7, 8].

Fibrinogen (Fgn) is a major plasma component and, besides its canonical hemostatic role in regulating platelet adherence and fibrin deposition, when, upon increased endothelial cell permeability, fibrinogen accumulates at stromal regions clotting into fibrin meshworks, it has also proven to have an angiostatic potential [9–11]. Fgn is a large protein (340 kDa) consisting of three pairs of heterogeneous polypeptides: the α-, β- and γ- chains, arranged into two outer D-domains and a central E-domain [12–15]. Physiologically, the digestion of fibrinogen by plasmin produces two D-fragments and a number of small peptides including β1-42 (the N terminus of the β-chain) and FgnE (a 50-kDa fragment consisting of the N-terminal regions of the α-, β- and γ- chains held together by disulphide bonds). In fact, two sub-fragments of FgnE were further generated and characterized. β43-63, a peptide that corresponds to the N terminus of the FgnE chain [16] and alphastatin, a 24-amino-acid peptide derived from the N-terminus of the α-chain of FgnE. Both peptides retain most of the FgnE anti-angiogenic effects *in vitro* and anti-vascular effects *in vivo*, without the cytotoxicity for activated ECs displayed by the FgnE.

Our group has previously shown that fibrinogen is a suitable macromolecular substrate for bothropasin, a Snake Venom Metalloprotease (SVMP) isolated from *Bothrops jararaca* venom and is one of the main proteolytic enzymes with selectivity for the fibrinogen α-chain [17]. Besides this, the fibrinogen-derived peptides released by bothropasin was identified, among them some known bioactive peptides were found [18]. A recent study using a human plasma-derived peptide library as substrate along with mass spectrometric technologies explored the peptide bond specificity of bothropasin and showed a clear preference for Leu at the P1' position, showed the consensus peptide XXGS-LLVL was derived with the Xs indicating no clear preference for any particular amino acid residue [19].

In the present work, we show that alphastatin-C, a new 14-aminoacid peptide consisting of a C-terminal fragment of the α-chain of Fgn generated by hydrolysis with bothropasin, is a potent inhibitor of bFGF induced EC activation *in vitro*. Alphastatin-C inhibits tumor angiogenesis and reduces melanoma tumor growth, inhibits the chick chorioallantoic membrane (CAM) angiogenesis, but surprisingly, stimulates the process of arteriogenesis, increasing postnatal pial collateral density in mice.

## RESULTS

### Alphastatin-C has anti-angiostatic effects on endothelial cells

**Figure 1:**
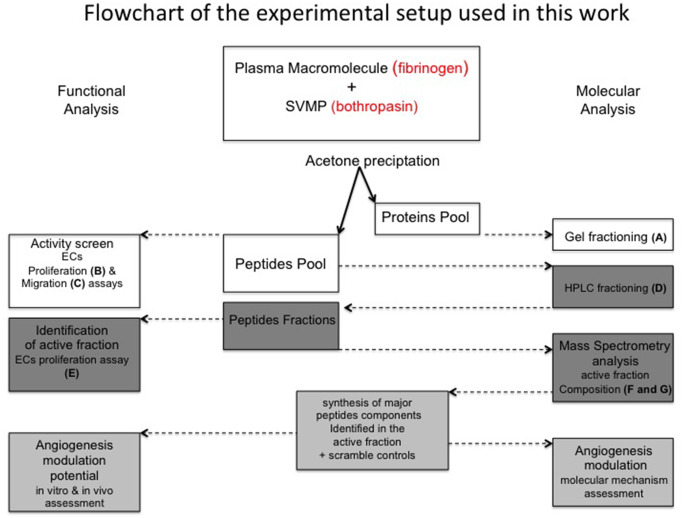
Flowchart of experimental procedures applied in this work. A plasma macromolecule (fibrinogen) was hydrolyzed by a SVMP (bothropasin). Acetone precipitation allowed to the recovery of large products (Proteins pool) and small products (Peptides pool). The strategies used for the functional (boxes in the left-hand side) and molecular (boxes in the right-hand side) analysis of fibrinogen-hydrolysis products are described. Letters indicate the panels in which the corresponding analyses are displayed in Figure 2.

**Figure 2:**
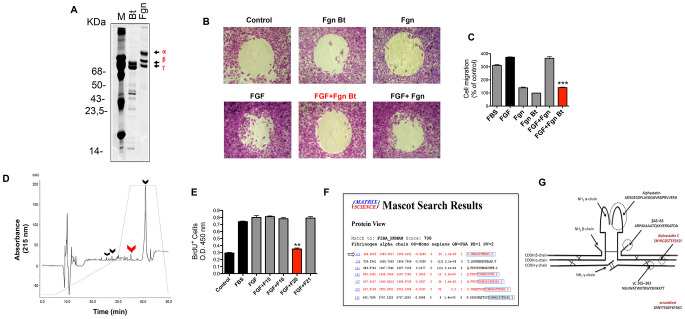
Alphastatin-C isolation and characterization. (**A**) SDS-PAGE shows that fibrinogen (Fgn) has its alpha chain preferentially cleaved by bothropasin (Bt). (**B**) Radius assay shows that FGF-induced (FGF) migratory action is inhibited in the presence of the peptide pool from bothropasin-hydrolyzed fibrinogen (FGF + Fgn Bt). The peptide pool from fibrinogen autolysis (Fgn) does not inhibit FGF (FGF + Fgn), and both isolated pools do not modulate cell migration (Fgn and Fgn Bt; **C**), quantitative analysis of Radius assay results in (B). (**D**) Reversed -phase chromatography analysis of Fgn Bt (full line) versus Fgn (dotted line) evidencing the peaks enriched in the former treatment, the peak 20 (red arrow) which proved to be effective on *in vitro* assays, and other differential peaks (15, 16, and 21) which are indicated by black arrows. (**E**) proliferation assay using BrdU incorporation to DNA demonstrating that peak 20 was the only one able to inhibit FGF action during this process (FGF + F20), basal DNA synthesis in DMEM (Control) is consistently increased by FGF (FGF) and 20% Fetal Bovine serum treatments (FBS), the remaining peaks did not affect FGF stimulation (FGF + F15, FGF + F16, FGF + F21). (**F**) Peak 20 was submitted to mass spectrometry analysis and the shortest RGD containing sequence (S.YNRGDSTFESKS.Y), among the most abundant hits (highlighted by the arrow), was identified. This peptide was named alphastatin-C, due its origin from C-terminal fibrinogen alpha chain, and it was selected for further synthesis and testing. (**G**) Scheme showing the location of fibrinogen-derived peptides with biological actions previously described in the literature: from fibrinogen alpha chain, alphastatin (black) and alphastatin-C (red) identified in the present work; from fibrinogen beta chain, 43-63; and from fibrinogen gamma chain, C 365-383.

To obtain the pool of peptides specifically generated by the hydrolysis of fibrinogen (Fgn) by bothropasin (Bt) we designed an experimental strategy (Figure 1) in which the peptide products of Fgn incubation in the absence (Fgn) or in presence of Bt (Fgn Bt) were recovered after acetone precipitation of the protein fraction. We first analyzed this resulting protein product by SDS-PAGE (Figure 2A) and confirmed that Fgn incubation with Bt led to the proteolysis of the Fgn α -chain (Fgn Bt, Figure 2A). As expected, low degradation was detected when Fgn was incubated in the absence of enzyme (Fgn control, Figure 2A). The supernatant from acetone precipitation containing the proteolysis-resulting peptide pool was quantified using fluorescamine reagent (data not shown) and 50 ng aliquots were assayed in cell proliferation and migration assays (Figure 2B and 2C). To analyze the modulatory activity of the peptide pool (Fgn and Fgn Bt) on proliferation, they were added to serum-starved HUVEC-CS cells either alone or in combination with FGF. As shown in Figure 2E, FBS and FGF could cause a significant increase (3.3 and 4 times, respectively) in BrdU incorporation when compared to non-stimulated control cells. FGF stimulation was almost completely abolished by concomitant addition of the bothropasin -generated peptide-pool (FGF in comparison to Fgn Bt + FGF), which, in turn, did not produce effects when administered alone (Fgn Bt). In contrast, control peptides (Fgn) were unable to produce any effects on cell proliferation when ministered alone (Fgn in comparison to control) or in combination with FGF (FGF + Fgn compared to FGF). The investigation of Fgn-derived peptide pools (Fgn and Fgn Bt) modulatory activity on EC migration (Figure 2C) showed no significant effect on cell migration since the coverage of the central spots in the Radius™ assay, 20 h after stimulation, was not distinguishable from that observed in non-stimulated cells (control). FGF was used as a positive control, inducing significant levels of migration. When FGF was combined with the control pool (FGF + Fgn) its stimulatory effect was not affected, but the combination of FGF with the bothropasin-generated pool (FGF + Fgn Bt) significantly inhibited FGF migratory activity.

Our next step was to fractionate the two peptide pools (Fgn Bt and Fgn) by reserve phase chromatography on HPLC system (RP-HPLC). Our results (Figure 2D), show that four eluted sub-fractions (F15, F16, F20, and F21) were significantly enriched in the Fgn Bt sample, and those were collected for BrdU incorporation activity test. As shown in Figure 2E the subfractions were tested for their ability to abolish FGF triggered BrdU incorporation. Positive controls (FBS and FGF) stimulated BrdU incorporation levels when compared to untreated cells (control). When each subfraction was combined to FGF, only subfraction 20 could reverse the stimulatory effect of FGF (FGF+ F20). We then proceeded to the analysis of F20 components by ESI-Q-TOF mass spectrometry (Figure 2F). The peptides identified in F20 were searched against human fibrinogen alpha-chain using a MASCOT search tool and the most abundant peptides identified are displayed in Figure 2F. Based on these results we designed and synthesized a peptide containing the minimal core present in four of the major peptides as highlighted in Figure 2F. We called this peptide alphastatin-C due to its position at the C-terminal region of the Fgn alpha-chain and also to differentiate it from a previously described Fgn-derived peptide localized in Fgn N-terminal region named alphastatin. As a negative control, we synthesized a scrambled (SCR) version of alphastatin-C with the same length and amino acids composition but with a disruption of the RGD motif. A schematic display of the relative localization of alphastatin-C and other previously described Fgn-derived peptides is shown (Figure 2G). Thus, we have identified a novel peptide selectively generated by the proteolytic cleavage of human fibrinogen by the SVMP bothropasin. The HPLC subfraction containing this 14-amino acid peptide as its major component proved to efficiently inhibit FGF-stimulated mitogenic activity.

### Alphastatin-C inhibits the bFGF-triggered angiogenic activity of endothelial cells *in vitro* as monitored by proliferation and migration assays

**Figure 3:**
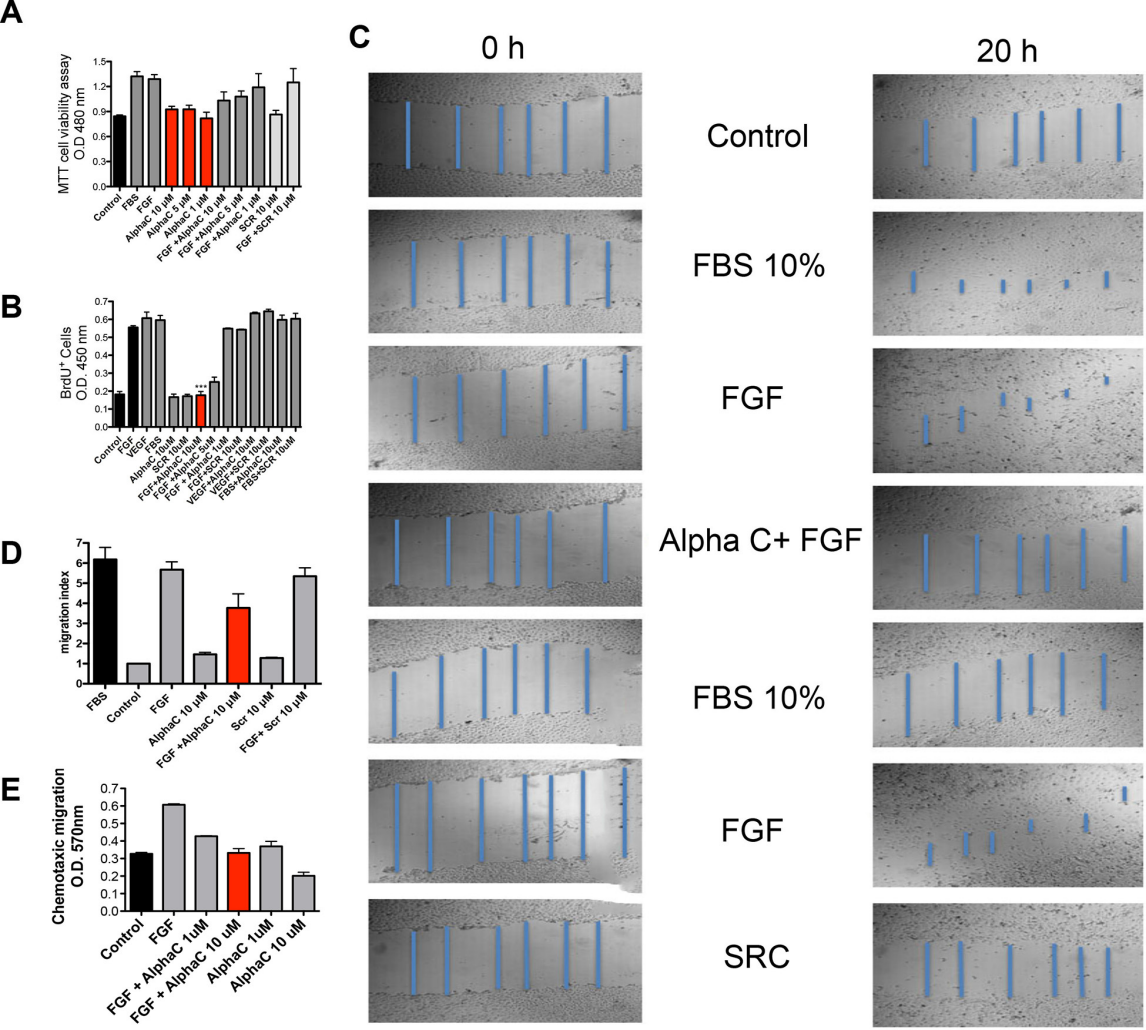
Effects of alphastatin-C on the proliferation, viability, chemotactic migration and chemokinetic migration of HUVEC-CS. (**A**) For cell viability evaluation, exponential cultures of HUVEC-CS were treated for 24 h with 20% Fetal Bovine Serum (FBS), bFGF 20 ng/mL (FGF), indicated concentrations of alphastatin-C (alpha-C), the combination of bFGF and alphastatin-C (FGF + alpha-C), and 10 μM scrambled peptide alone (SCR) or combined to bFGF (FGF + SCR), after which MTT assay was carried out. None of the peptides displayed toxicity at tested concentrations range. (**B**) cell proliferation was evaluated by 6 h BrdU incorporation after 12 h stimulation with the indicated experimental treatments, besides the treatments in (A), VEGF was used alone (VEGF), combined with 10 μM alphastatin-C (VEGF+alpha-C), or with 10 μM Scrambled peptide (VEGF + SCR). (**C**) a wound-healing assay displaying scratch images at 0 h and 20 h time points after indicated treatments, in blue, scratch measurements used to determine the migration indexes (procedure detailed in Materials and Methods) shown in (**D**). (**E**) chemotaxic cell migration by the Boyden chamber migration test, FGF acts as chemoattractant (FGF compared to Control) which is inhibited in a dose-dependent manner by simultaneous treatment with alpha-C (FGF + alpha-C 1 μM, and FGF + alpha-C 10 μM), whereas alpha-C peptide alone does not attract endothelial cells (alpha-C 1 μM, alpha-C 10 μM). We consistently observe (all shown assays) that the treatment with 10 μM alpha-C can inhibit the stimulatory action (proliferation, chemokinetic migration and chemotactic migration) triggered by bFGF 20 ng/ml, whereas the scrambled peptide has no such effect.

The next step was to test the synthetic peptides for their effect on ECs migration and proliferation and to verify whether alphastatin-C could recapitulate the inhibitory activity of the whole RP-HPLC subfraction on FGF-induced processes. Figure 3A shows representative toxicity results using the MTT reagent. None of the peptide treatments at any of the tested doses produced significant toxicity or proliferative activity (alphastatin-C 10, 5, and 1 μM and SCR 10 μM compared to control) in endothelial cells. We verified that at 10 μM concentration peptide alpha-C also produced a significant decrease in FGF-induced cell proliferation (FGF + alphastatin-C compared to FGF). At this same concentration, the scrambled peptide (SCR) had no significant action on MTT assays, being indistinguishable from control and non-effective when combined with FGF.

BrdU incorporation is a direct index of mitogenic activity providing different information from the physiological assessment in MTT assays. We have therefore chosen to perform the former as a complementary procedure to the later. Representative results are shown in Figure 3B. No mitogenic activity could be observed upon treatment with 10 μM alphastatin-C, 10 μM SCR or in the absence of stimulation (control). The positive controls (FGF, VEGF, and FBS) induced a significant increase in BrdU incorporation. This effect was inhibited by alphastatin-C in a dose-dependent manner, completely abolished with a 10 μM dose, partially decreased with a 5 μM dose, and unaffected with 1 μM. The stimulatory activities of VEGF and FBS remained unaltered in combination with 10 μM alphastatin-C or 10 μM SCR, which strongly suggests that the inhibitory activity of alphastatin-C was specific to FGF-triggered stimulation.

The potential to stimulate cell migration of 10 μM alphastatin-C, SCR, FGF, FBS and each peptide alone and in combination to FGF was assessed in chemokinetic assays, in which the tested molecules were added as a homogeneous solution to scratched monolayers of adhered HUVECs (Figure 3C). 24 h after stimulation, FGF and FBS produced a strong (5 to six-fold by comparison with the control) stimulation in ECs migratory activity. However, 10 μM alphastatin-C and 10 μM SCR did not alter the basal migratory index presented by non-stimulated cells (control). Upon addition of 10 μM alphastatin-C, the FGF stimulatory effect was reduced by 40%, whereas the presence of 10 μM SCR in combination with FGF had no significant effect on its migratory stimulating activity, as shown by the quantitative results produced by control normalization of optical density measurements and shown as migration index (Figure 3D). Endothelial cells are particularly sensitive to growth factor gradients; the directional cell migration towards increasing concentrations of a given molecule can be measured in chemotaxis assays (Figure 3E). FGF (20 ng/mL) acts as a chemoattractant, doubling the number of cells that migrated to the FGF-containing compartments through the 8 μm pores in Boyden chamber wells (FGF compared to control) 20 h after the assays started. When cells were treated with 1 μM and 10 μM alphastatin-C at initial times their migratory activity towards FGF was inhibited in a dose-dependent way, with the lower dose producing an inhibition of about 30%, and the higher dose inhibiting FGF-induced chemotaxis by 50%. In comparison to basal levels of directional migration, when no chemoattractant is added to the migratory chamber (control), the incubation in the presence 1 μM alphastatin-C had no effect, whereas 10 μM alphastatin-C produced a 30% percent inhibition. Thus, we established that the RGD-containing peptide alphastatin-C selectively blocked FGF induced proliferation, mitogenic activity, chemokinetic migration, and chemotaxis. The critical concentration producing maximal inhibitory effects and lacking toxicity is 10 μM alphastatin-C.

### Alphastatin-C blocks endothelial cell signaling by bFGF as monitored by early, mid and late-triggered signal transduction hallmarks

**Figure 4:**
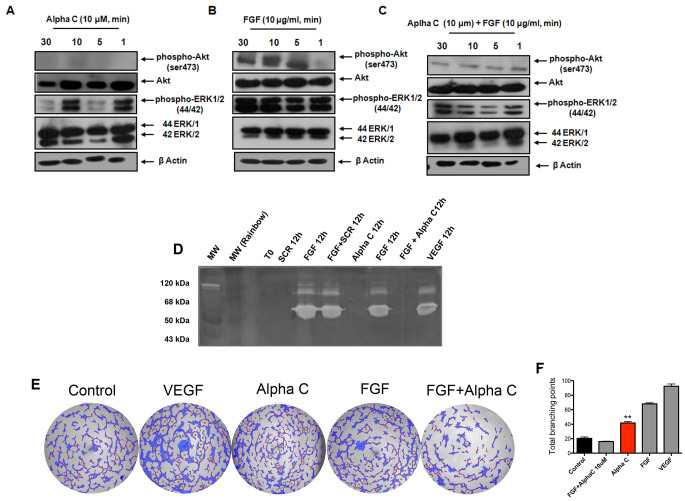
Alphastatin-C blocks ERK and Akt activation by FGF and inhibits FGF-triggered MMPs secretion and tubulogenic activity. HUVEC were cultured and treated with 10 μM **alphastatin-C**, 20 ng/ml FGF alone, or 10 μM alpha-C combined with 20 ng/ml FGF for 30, 10, 5 and 1 minutes. (**A**–**C**). The expression of p-Akt, Akt, p-ERK1/2 and ERK1/2 were evaluated by Western blots. Of note, β-Actin was used as loading control. Additionally, the effects of alpha-C on MMP-2 and MMP-9 activities on the conditioned medium of 12 h treated cell cultures were tested for gelatinolytic activity by gelatin zymography (**D**). There was a strong reduction of MMP-2 and MMP-9 activities triggered by FGF in response to alpha-C treatment *in vitro* (FGF + alpha-C 12 h) when compared to FGF alone (FGF 12 h), whereas the treatment with 10 μM scrambled peptide had no effect over FGF action (FGF + SCR 12 h). alpha-C and Scrambled peptide (alpha-C 12 h and SCR 12 h) did not induce proteolytic activity, and 40 ng/ml VEGF was used as positive control. In panel (**E**) tubulogenic activity was evaluated by plating HUVEC on matrigel. alpha-C + FGF inhibits capillary-structure of HUVEC on the matrigel. After 24 h of incubation in the presence of DMEM (Control), 40 ng/ml VEGF, 10 μM alpha-C, 20 ng/ml FGF, or the combination (FGF + alpha-C), images were documented and total branching points were quantified using WimTube Key Metrics Program (**F**), showing that alpha-C significantly inhibits FGF-induced sprouting angiogenesis (total branching in FGF + alpha-C versus total branching in FGF).

Given the efficacy of alphastatin-C as an antagonist of FGF on cell proliferation and motility, we decided to further investigate the molecular mechanisms involved. We chose to monitor the capacity of alphastatin-C to modulate three downstream molecular pathways typically engaged by FGF signal transduction, i.e.: I) Activation of the MAP kinases ERK 1/2 and Akt; II) induction and secretion of MMPs to the cell cultures supernatants; and III) The stimulation of capillary-like tube formation in Matrigel. We first studied the expression and phosphorylation (at Thr202/Thr204) of ERK 1/2, which is strongly activated during the angiogenic program. The effects of the treatments of serum-starved endothelial cells with FGF, alphastatin-C or with the combination of both (Figure 4A–4C) at different times after stimulation were analyzed by Western blots. Addition of alphastatin-C alone, FGF or the combination (alphastatin-C + FGF) did not affect ERK 1/2 expression levels as shown by constant levels of total proteins (ERK/1 and ERK/2 lanes in Figure 4A, 4B, and 4C).

Nevertheless, the phosphorylation of ERK1/2 was differently modulated by the treatments, is strongly induced by FGF as soon as 1 min up to 30 min [phosphor-ERK1/2 (44,42), Figure 4B]. This induction was strongly inhibited in the combined (alphastatin-C + FGF) treatment at all tested times [phosphor-ERK1/2 (44,42), Figure 4C], whereas alpha-C treated cells showed an oscillatory pattern of ERK1/2 phosphorylation, with increased levels at times 1 min and 10 min, returning to basal levels at times 5 minutes and 30 minutes [phosphor-ERK1/2 (44,42), Figure 4A]. We next studied the effect of alphastatin-C on the Akt pathway, because the Akt phosphorylation (Ser473) induced by FGF and other growth factors is involved in endothelial cell survival, which is modulated during angiogenesis. Similar to ERK, no significant modulation of Akt expression levels were observed in alphastatin-C, FGF, or combined treatments as monitored by constant protein levels along the four tested time points (Akt lanes in Figure 4A, 4B, and 4C).

Our results indicate that the alphastatin-C treatment leads to non-significant Akt activation at any tested time [phosphor-Akt (ser473) lane, Figure 4A], FGF induced Akt phosphorylation from five minutes up to 30min after stimulation [phosphor-Akt (ser473) lane, Figure 4B], and the combination of FGF and alpha-C significantly reduced the FGF-triggered Akt phosphorylation at all monitored time points [phosphor-Akt (ser473) lane, Figure 4C].

Another signature of FGF signaling in endothelial cells is the induction of MMPs secretion to the growth medium after stimulation, MMP-2 and MMP-9 [20]. Therefore, we decided to examine the effects of alphastatin-C on the activity of the MMPs. Serum-starved HUVEC-CS were treated with 10 μM alphastatin-C or 10 μM SCR in the presence or absence of FGF for 12 h; the media were collected, concentrated and tested for gelatinolytic activity by gelatin zymography. As shown in Figure 4D, MMP-2 and MMP-9 activities by FGF (FGF 12 h) were significantly reduced in response to alphastatin-C (FGF + alphastatin-C, 12 h) but were unaffected by the SCR treatment (FGF + SCR, 12 h), when alphastatin-C and SCR peptides were added to cultures in absence of FGF, both peptides were unable to induce gelatinolytic activities within a 12 h period.

The formation of capillary tube structures in Matrigel is also a well-known response of ECs to FGF [21]. The modulatory effect of alphastatin-C on tubulogenic activity in FGF stimulated HUVEC-CS was investigated. Representative results shown in Figure 4E demonstrated that in comparison to control (basal medium with no factor addition), either VEGF or FGF could produce significant levels of tubule formation, as shown in the quantification graphic compiling results from three independent experiments (Figure 4F). In contrast, alphastatin-C alone produced no significant effect on the number of formed tubules, after 48 h of stimulation, however, when it was combined to bFGF, its stimulatory action was inhibited.

In summary, these data show that at least three distinct stages of the endothelial cell response to bFGF were efficiently inhibited by concomitant treatment with 10 μM alphastatin-C. The transduction of the bFGF signal requires the early phosphorylation of Akt and ERK1/2, the intermediate induction of MMPs, and the late formation of capillary-like tubules. These steps of endothelial cell response were efficiently inhibited by concomitant treatment with FGF and 10 μM alphastatin-C.

### Inhibitory activity of alphastatin-C disrupts FGF-induced integrin αVβ3 clustering and requires its co-localization with this integrin

Integrins are heterodimeric transmembrane cell surface receptors linking the actin cytoskeleton to the extracellular matrix, which transduce signals bidirectionally across the cell membrane. In this context, it was important to investigate whether alphastatin-C could compete with specific extra-cellular molecules for association with their canonical integrin ligand.

**Figure 5:**
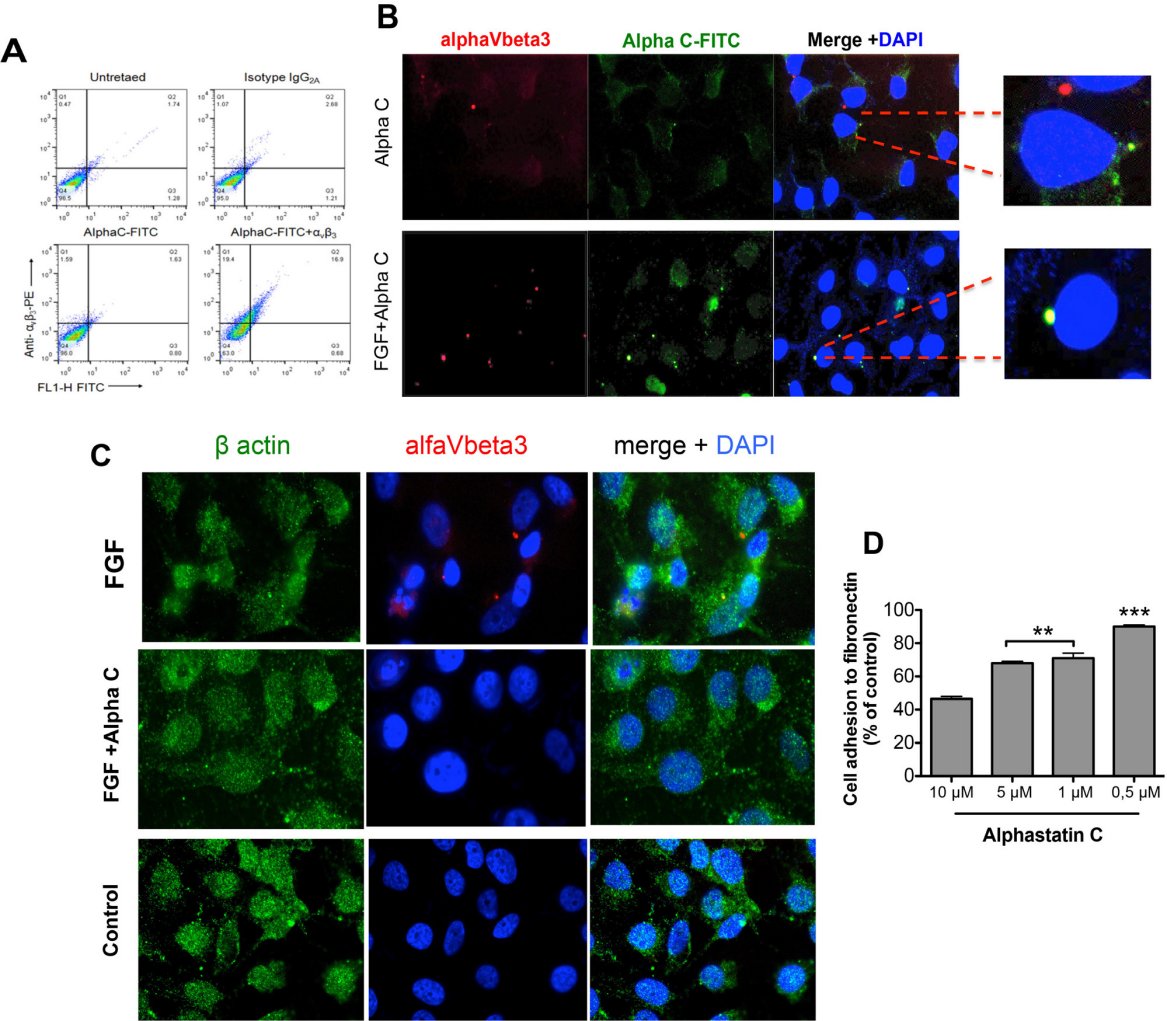
Alphastatin-C uncouples FGF-induced integrin V3 clustering and co-localizes with this integrin in the presence of bFGF. HUVEC-CS were seeded and treated with 10 μM FITC-alpha-C for 12 h. The fixed cells were then stained with PE-labeled anti-human antibody or isotype control IgG2, and anti-V3 and analyzed by flow cytometry (**A**). Alternatively, cells were cultured in fibronectin-coated slides in presence of 10 μM alpha-C-FITC peptide, stained for V3 (PE) and DNA (DAPI) and analyzed by Fluorescence microscopy, panel (**B**) shows that alphastatin-C co-localizes with the integrin only in the presence of FGF (FGF+alpha-C Merge +DAPI insert compared to alpha-C Merge + DAPI insert). Additionally, cells grown in fibronectin-coated slides were treated for 1 h with DMEM (control), 20 ng/mL FGF alone (FGF), or in the presence of 10 μM alphastatin-C (FGF + alpha-C) and immunostained for actin (green), αVβ3 integrin (red) and DNA (blue), the merged images show that whereas FGF induced integrin clustering in cell membrane, this response was abolished by the presence of alpha-C (**C**). Finally, upon incubation with increasing concentrations of alphastatin-C, ranging from 0.1 μM to 10 μM, EC adhesion to Fibronectin was inhibited in a dose-dependent manner (**D**).

A series of experiments were conducted to determine the effect of alphastatin-C on the FGF-induced α_V_β_3_ clustering, since α_V_β_3_ is the main fibronectin ligand in endothelial cells. Figure 5A and 5B show flow cytometry analysis and fluorescent microscopy images in which ECs were grown in fibronectin-coated dishes and labeled by immunofluorescence using antibodies against β-actin and α_V_β_3._ When serum-starved cells were stimulated for 1 hour in the presence of 20 ng/mL of FGF, clusters of α_V_β_3_ were detected in cells membranes. On the other hand, the concomitant incubation with 20 ng/mL FGF and 10 μM alphastatin-C completely abolished the formation of the previously detected α_V_β_3_ clusters, restoring a condition similar to that observed in controls, when cells were kept in serum-free medium (data not shown).

Next, given the strong evidence supporting a direct interaction of FGF with α_V_β_3_ [22] we have chosen to verify whether alphastatin-C would co-localize with the integrin and prevent FGF response by disrupting the FGF/α_V_β_3_ interaction. To this end, we treated serum-starved cells with 20 ng/mL FGF in the presence of 1μM FITC-conjugated alphastatin-C (non-inhibitory concentration), or 10 μM FITC-conjugated alphastatin-C (inhibitory concentration), or only alphastatin-C at the higher concentration and proceeded to immunostaining for α_V_β_3._ As shown in panel (Figure 5B), in the absence of FGF, there is no superposition between alphastatin-C and α_V_β_3_ staining_._ However, when administered in the presence of FGF; the FITC-labeled peptides co-localized with α_V_β_3_ integrin. Not surprisingly, the results presented in Figure 5C, monitoring the fluorescence associated with cells stained for α_V_β_3_ with a PE-conjugated antibody, and treated with 20 ng/mL FGF in the presence of 10 μM FITC- alphastatin-C, show that the same cell population was positive for both markers. It indicates that only α_V_β_3_ positive cells could directly interact with alphastatin-C. alphastatin-C, ranging from 0.1 μM to 10 μM, EC adhesion to Fibronectin was inhibited in a dose-dependent manner (Figure 5D). In contrast, the presence of alphastatin-C did not significantly affect the adhesion of ECs to other substrates i.e., collagen type I, collagen type IV, and laminin (data not shown). Taken together, these results suggested that the inhibitory concentration of 10 μM alphastatin-C inhibited endothelial cell adhesion to fibronectin, and abolished FGF-induced α_V_β_3_ clustering. Under the same conditions, alphastatin-C seemed to directly interact with integrin α_V_β_3_ as outlined by the qualitative and quantitative data.

### Alphastatin-C has an inhibitory effect on *in vivo* angiogenesis

Once we established the molecular basis for alphastatin-C inhibitory actions in cells, we proceeded to the investigation of its potential anti-angiogenic effect *in vivo.* For this purpose, we have chosen two distinct models, which provided alternative contexts to the assessment of new vascularization, i.e.: 1) the chick CAM assay that allowed the evaluation of the peptide’s effect on embryonic angiogenesis; 2) a model of dorsal melanoma tumor development allowing the evaluation of potential effects on tumor progression.

### Alphastatin-C inhibits angiogenesis in CAM assays

**Figure 6:**
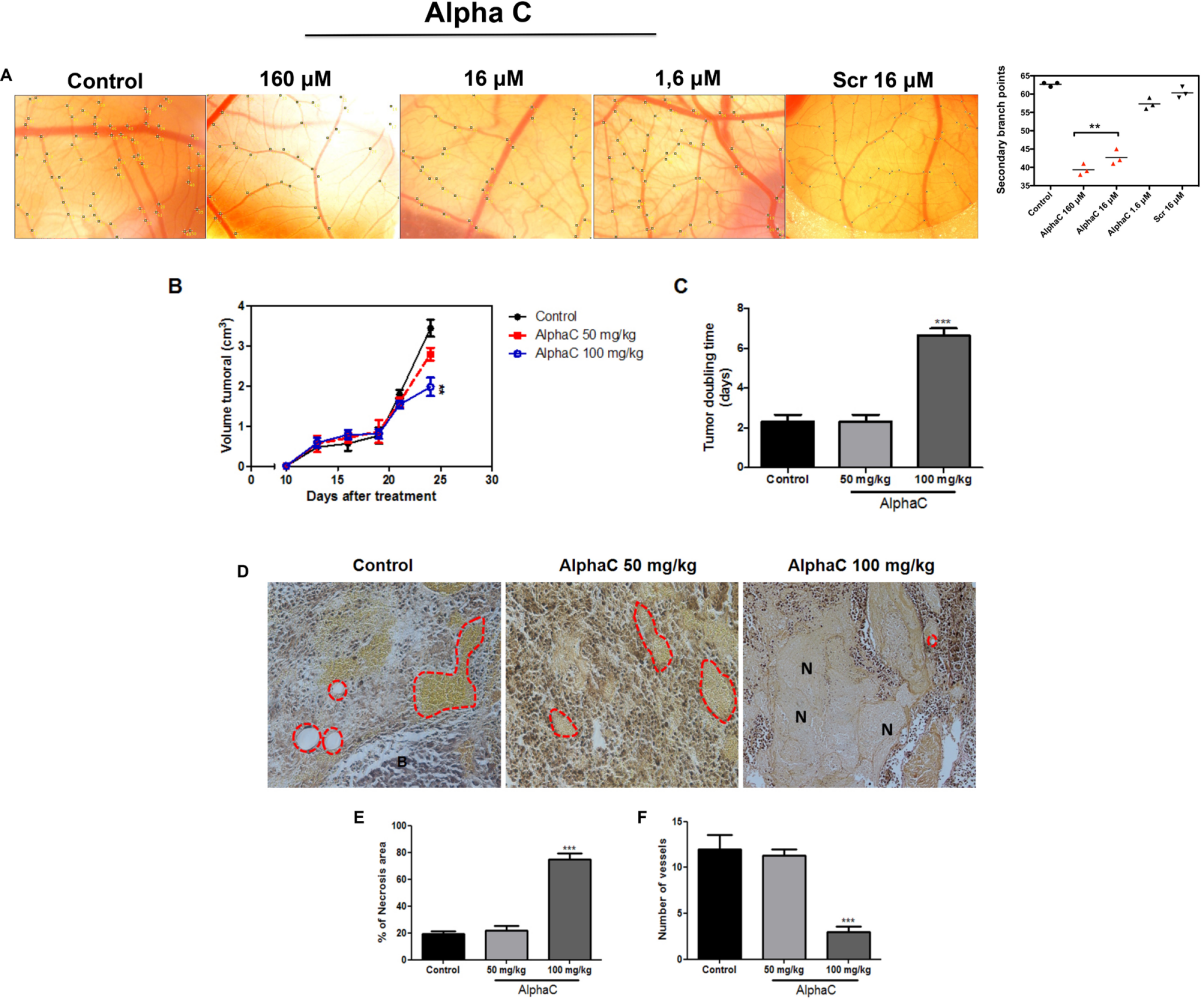
Alphastatin-C inhibits angiogenesis *in vivo*. (**A**) Bright field micrographs illustrating the chick CAMs at embryonic day 12 (E12) after application of the indicated factors inside a silicone ring at E10. Vascular plexus exposed to PBS-0.1 % BSA (control) displays a normal aspect and a mean of 62 secondary branch points per field (graphic in the right shows mean values of the quantification of two eggs in three independent experiments), alphastatin-C applied in three doses (160 μM, 16 μM, and 1.6 μM) induces a dose-dependent inhibition of sprouting angiogenesis with significant effects (38–45 secondary branches per field) at the higher doses 160M and 16M. Scrambled peptide (SCR) was tested at the intermediary dose (16 μM) and produced no significant sprouting angiogenesis modulation (**B**). Antitumor effect of alphastatin-C against subcutaneous melanoma in syngeneic mice. Animals (n = 5 per group) were subcutaneously injected with 5 × 10^4^ cells and treatment started on day 12, after the tumor has reached 100 mm^3^, with daily doses of 50 and 100 mg/kg alpha-C or vehicle (PBS). Intraperitoneal therapy was conducted during 15 consecutive days. Bars indicate means ± SD. (**C**) Additionally, alpha-C presented a significant increase in tumor doubling time. (**D**) Representative photomicrographs of tumor sections stained with hematoxylin-eosin (magnification, 100×). The control showed the large density of tumor cells while animals treated with 50 and 100 mg/kg alpha-C presented an increase of the necrotic area and consequently reducing the number of vessels. (**E**) Quantification of necrotic area and number of vessels. All values are expressed as averages ± S.D. of multiple determinations. **p < 0.01 and ***p < 0.001compared with untreated statistically significant.

Experiments were performed using fertilized chick eggs to monitor alphastatin-C effects on developmental angiogenesis between 10 and 13 embryonic days (E10 and E13). In Figure 6A, we show representative bright fields from E13 CAMs that were treated at E10 and E12 with PBS, 160 μM alphastatin-C, 16 μM alphastatin-C, 1.6 μM alphastatin-C, or 160 μM Scramble peptide. The quantification of the number of secondary branch points per field (Figure 6B) clearly showed that at the higher and intermediate doses, alphastatin-C significantly inhibited CAM vascular sprouting, by factors of 75% at 160 μM and 70% at 16 μM when compared with PBS-treated eggs. The membranes exposed to lower doses of alpha-C (1.6 μM) and to 16 μM scrambled peptide did not have their branching activity by E13 significantly changed.

### Alphastatin-C promotes anti-tumor effects in mice bearing a dorsal melanoma tumor

To investigate whether the *in vitro* anti-angiogenic effects of alphastatin-C could be related to an *in vivo* antitumor activity, we evaluated the action of alphastatin-C in a well-established animal model (Figure 6B–6F). B16-F10 cells (5 × 10^4^/mouse) were injected into the right dorsal flank of C57BL/6J mice by subcutaneous injection. When the tumor reached approximately 100 mm^3^ the mice were randomly allocated (n = 10) to a group in which they were treated for 15 consecutive days with either 100 μL PBS (vehicle/control), alpha-C (50 mg/kg or 100 mg/kg) by intraperitoneal injections. Mice treated with 50 mg/kg alpha-C had smaller tumors (2.8 cm^3^ ± 0.12) than control (3,9 cm^3^ ± 0,3) although the difference was not significant. However, tumor volume in mice treated with 100 mg/kg alpha-C significantly decreased by 56% (1,75 cm^3^ ± 0,08; **p< 0.05), indicating that alphastatin-C had a potent antitumor activity (Figure 6B). Tumor doubling time is an important parameter to determine treatment efficacy. In agreement with the expressive decreased of tumor volume, the treatment with 100 mg/kg of alphastatin-C increased the tumor doubling time (6. ± 7, 5 days; **p< 0.05) by comparison with control mice (2 ± 2, 5 days) or mice treated with 50 mg/kg (2, 5 ± 2, 2 days) (Figure 6C). To further investigate whether the antitumoral activity of alphastatin-C was directly linked to the angiogenesis inhibition, the dorsal lesions were removed and histologically analysed after Verhoeff’s van Giesen stain. As shown in Figure 6D, tumor sections of control and mice treated with 50 mg/kg alpha-C showed many hemorrhagic areas with vessels anastomoses and rare foci of fibrosis. By contrast, the treatment with 100 mg/kg alphastatin-C increased necrotic areas (Figure 6E) and reduced the number of newly formed blood vessels (Figure 6F). Collectively, these findings indicated that the inhibition of tumor development by alphastatin-C could be associated with the inhibition of tumor blood vessels formation in a melanoma model, suggesting that necrosis could be an outcome of the impaired angiogenesis.

### Alphastatin-C stimulates post-natal brain arteriogenesis in mice

**Figure 7:**
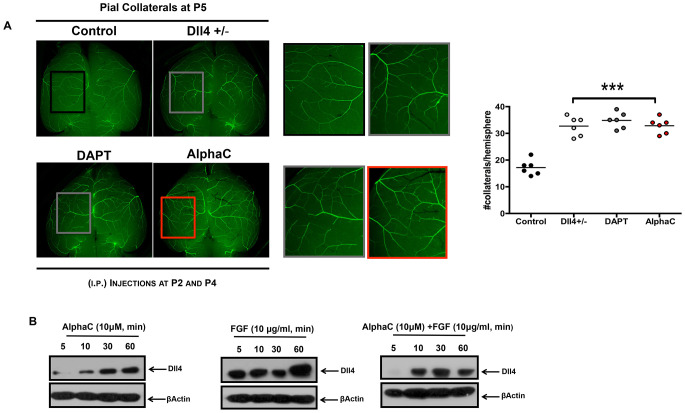
Alphastatin-C increases pial arteriolar formation *in vivo*. (**A**) Representative microphotographs of collateral arteriolar connections (arrows in magnified inserts) between MCA and ACA in P5 brains of wild-type Gja5eGFP/+ mice, Dll4 ± X Gja5eGFP/+mice, or animals treated with 100 DAPT mg/kg, or 100 mg/kg alpha-C by intraperitoneal injections at P2 and P4. Higher magnification of boxes (inserts with matching colors) show an increased number of arterioles in Dll4 +/-, DAPT treated and alpha-C treated brains as compared to wild-type non-treated mice. The number of collateral connections per hemisphere per mouse is shown for each treatment (six animals in two independent experiments). (**B**) *In vitro*, HUVEC were treated with 10 μM alpha-C, 10 ng/mL FGF or 10 μM alpha-C + 10 ng/mL FGF for 5, 10, 30 and 60 min. Afterward, cells were lysed and total protein isolated. Then, cell lysates were electrophoresed and immunoblotted for Dll4. -actin antibody was used as loading control. FGF induced an increase in Dll4 levels (FGF all time points) is inhibited in presence of alphastatin-C (alpha-C + FGF all time points), the treatment with alpha-C alone displays basal Dll4 levels.

The process of arteriogenesis is based on the growth and remodeling of arteriole-to-arteriole anastomoses [22–25] and differs from classical capillary sprouting described during angiogenic processes. Considering the distinct features of collateral growth, we evaluated the effects of alphastatin-C on the postnatal development of collateral vessels in the brain, which typically link the middle cerebral artery (MCA) and the anterior cerebral artery (ACA) in pial circulation. Here, we used transgenic CD1 expressing EGFP controlled by the arterial Gja5 (connexin 40) promoter [26] that permit to visualize fluorescent collateral vessels in the pial circulation of P5 mice. The intraperitoneal administration of 100 mg/kg alphastatin-C at P2 and P4 significantly increased the number of MCA/ACA anastomosis in treated brains as compared to PBS injected animals (Figure 7A). Since Dll4 ablation, either by pharmacological or by genetic means, also triggers an increase in collateral vessels number [27], we compared the effect of alphastatin-C with the role of Dll4 inhibition in DAPT (a gamma-secretase inhibitor) injected mice (100 mg/kg at P2 and P4), and in Dll4 ^+/-^defiecient CD1 mice that were crossbred with the arterial EGFP-expressing mice. As expected, both conditions produced an increase in pial collateral vessels number (Figure 7B).

Considering these previous results, we tested whether alphastatin-C would affect Dll4 protein levels in cell cultures. ECs were serum-starved for 48 h and treated with either alphastatin-C (10 μM), FGF (20 ng/mL), or the combination of both agents for 5 min, 30 min and 1 h. Our data showed that FGF-treated cells presented Dll4 induction upon 5 minutes stimulation, and Dll4 remained at detectable levels up to 1 h after treatment (Figure 7A). In alphastatin-C-treated cells we only could detect Dll4 protein after 10 min, the signal intensity progressively increased in the following time points (0, 30, and 60 min), but remained lower than in FGF-treated cells always. Finally, in cells submitted to the combined treatment (alphastatin-C + FGF), the stimulatory effect of FGF was delayed (appeared only after 10 min) and inhibited (band intensity was lower than in FGF alone treatment for all time points). These results showed that Dll4 is another molecular marker for the antagonism between FGF and alphastatin-C *in vitro* and a likely intermediary of alphastatin-C arteriogenic action *in vivo*.

## DISCUSSION

Alphastatin-C is a 14 amino acids peptide generated by the proteolysis of human fibrinogen by the SVMP bothropasin. It is a C-terminal fragment of the Fgn alpha chain, whose biological role has not, to our knowledge, been characterized before. Here we show that alpha-C is: (1) an efficient and selective blocker of FGF-induced endothelial cell proliferation, migration and chemotaxis; (2) abolishes the transduction of the FGF signal as monitored by early, mid, and late events; (3) a selective competitor of EC adhesion to fibronectin that is able to inhibit FGF-induced α_V_β_3_ clusterization in adhered cells, and colocalizes with this integrin in the presence of FGF; (4) a potent inhibitor of *in vivo* angiogenesis in embryonic and tumoral conditions as verified by experiments in two distinct model systems; (5) an inducer of post-natal cerebral arteriogenesis in mice that phenocopies the genetic and pharmacologic ablation of Dll4.

FGF is a canonical angiogenic molecule, which is required in EC cultures to support cell proliferation, prevent apoptosis [28] and promote cell migration [29]. The binding of FGF to fibrinogen was previously shown to potentiate FGF stimulatory activities [30]. Having generated synthetic alphastatin-C, we investigated its effects on FGF-induced proliferation, migration and chemotaxis in endothelial cells. We verified that alphastatin-C acts as a potent and selective FGF antagonist in all these tested conditions. A possible alphastatin-C cytotoxicity was ruled out based on MTT incorporation assays that showed that even though the peptide abolished EC proliferation induced by FGF, it did not alter cell viability below control (untreated cells) levels.

It is well established that FGF signal transduction promotes the phosphorylation of ERK and Akt at specific residues [31–33] as early events in the activation of the MAPK pathways. alpha-C significantly inhibits these two events. In this regard, the fact that it has been previously described that FGF, but not VEGF, requires both events for its proangiogenic action agrees with the FGF selective blockage by alphastatin-C [34]. The secretion of matrix metalloproteinases and the building of tubular structures are additional steps in endothelial cells activation by FGF that are also efficiently inhibited by alpha-C.

Our next step was to verify whether alphastatin-C would interfere with the adhesion endothelial cells to specific substrates, which are main components of alternative vascular beds. Our results show that alphastatin-C competes with fibronectin for cell adhesion but has no significant effect on its binding to collagen, laminin, and vitronectin. As the interaction between fibronectin and endothelial cells is mainly mediated by α_V_β_3_, we investigated the effect of alphastatin-C on its activation. The immunofluorescent labeling assays we performed clearly show that alphastatin-C impairs FGF-triggered α_V_β_3_ clustering in EC membranes. Besides, using Fitc-conjugated alphastatin-C we established by FACS that the peptide selectively binds to α_V_β_3_-expressing cells. We also established, by IF labeling that alphastatin-C co-localizes with this integrin only in the presence of FGF. These results are supported by data in the literature showing a direct interaction between FGF and α_V_β [21], suggesting that alphastatin-C could directly interact with FGF and, even though this interaction did not disrupt FGF binding to the integrin (Figure 5D), it somehow made it ineffective, abolishing the activation of endothelial cells by FGF. Interestingly, the work of Sahni [30] and collaborators determined that Fibrinogen binding to FGF enhances its proliferative activity, which brings up the possibility that alphastatin-C competes with fibrinogen for the interaction with FGF, inhibiting rather than potentiating FGF-triggered endothelial cells signaling.

Once we had examined the cellular and molecular mechanisms of alphastatin-C targeted inhibition, we evaluated it is *in vivo* anti-angiogenic potential. The picture emerging from the *in vivo* experiments is that alphastatin-C has marked effects on embryonic angiogenesis. The peptide was also an efficient inhibitor of tumor neovascularization in a murine melanoma model, significantly reducing tumor volume and number of vessels, while significantly increasing tumor doubling time and necrotic area. In these two *in vivo* models, the fact that alphastatin-C drastically inhibited new blood vessel formation without effect on the underlying vasculature agrees with the lack of toxicity of the peptide observed in cultured cells by MTT assays. It also rules out the possibility of a nonspecific deleterious effect on pre-existing mature capillaries. Our results suggest that the alphastatin-C inhibitory activity can be specifically targeted to activate endothelial cells without affecting stable vasculature *in vivo.* These results are corroborated by the fact that, *in vitro*, alphastatin-C does not produce any inhibitory effect on the basal levels of proliferation or migration of non-stimulated endothelial cells.

Since alphastatin-C proved to be a suitable inhibitor of sprouting angiogenesis and of tumor neovascularization *in vivo*, we found it interesting to test the peptide effects in a third angiogenic context, the development of collateral vessels. Interestingly, alphastatin-C acted as a stimulating agent of this process, also known as arteriogenesis, that bears important differences in comparison to the former two tested, as it requires selective remodeling and pruning of previously existing arterial connections. The increase in pial collateral vessels number induced by early post-natal alphastatin-C administration mimics the effect of Dll4 ablation in this model, [35] which led us to monitor the effect of alphastatin-C treatment on Dll4 levels in cultured cells. Not surprisingly, we found that the peptide significantly reduces Dll4 induction by FGF. Interestingly, it has been previously described that bFGF does not significantly upmodulate arteriogenesis after femoral artery ligature in rats [36], which could explain the stimulatory effect of bFGF blocking by alphastatin-C in the context of collateral formation, as opposed to its inhibitory action on angiogenesis. Taken together, the *in vivo* results concerning alphastatin-C are in agreement with many facts previously described in the literature, e.g., Dll4 consistent inhibition during tumor angiogenesis; [37] impairment of intravascular fibrinolytic activity in tumors; [38] the observation that FGF overcomes sunitinib (a VEGF inhibitor) inhibition to support tumor growth [39]; the marked distinctions in the response of pericyte-lined tubes versus angiogenic tubes to proteolytic challenges; [4] the presence of increased levels of fibrinogen in vessels microenvironment in pathological conditions; [40, 41] the presence of fibrinogen acting as vessel wall protection from proteolysis [42].

In conclusion, a heterologous metalloproteinase, the SVMP bothropasin, was successfully used to unravel a new angiostatic peptide named alphastatin-C, generated by the proteolytic cleavage of fibrinogen α-chain. This strategy, used here for the first time, could become a general procedure in the search of new statins. Besides, alphastatin-C blocks the events stimulated by FGF *in vitro*, it inhibits FGF-triggered proliferation, migration, chemotaxis, integrin clustering, ERK and Akt activation in endothelial cells. Most importantly, alphastatin-C impairs tumor vascularization and reduces developmental angiogenesis *in vivo*. At the same time, alphastatin-C acts as a stimulatory agent in pial collateral growth, which is compatible with its inhibitory effect over Dll4 levels *in vitro*. Dll4 is a transmembrane ligand of Notch receptors that is selectively expressed in arterial endothelial cells and angiogenic tip cells during development. Among many other actions, it has been shown that Dll4-loss of function mice show reduced vascular branching during embryogenesis. Besides, Dll4-Notch signaling determines the de novo formation of arterial collateral networks and arterial function in mouse ischemia models. These results are in agreement with the inhibitory effect of alphastatin-C in artery development and its stimulatory action on the formation of collateral connections. Though other active fibrinogen-derived active peptides have been previously characterized based in rational design, our work is also the first to show the effects of this RGD-containing peptide that could potentially be generated by enzymatic proteolysis in the physiological context. Further studies are required to address this possibility as well as the structural details of FGF, Fgn, alphastatin-C, and α_V_β_3_ interactions.

## MATERIALS AND METHODS

### Peptides, proteins, antibodies, drugs

Synthetic alphastatin-C (alpha-C) and scrambled peptides (SCR) were prepared in automated bench-top simultaneous multiple solid-phase synthesis (PSSM 8 system; Shymadzu, Kyoto, Japan) using solid phase peptide synthesis by the Fmoc procedure. Briefly, sequential couplings of protected amino acids were performed with HOBt, TBTU, and NMM on l-proline-2-chlorotrityl resin (Novabiochem, Gibbstown, NJ, USA). Fmoc cleavage was performed with 50% morpholine (v/v) in DMF. The resin-bound peptides were cleaved/deprotected with TFA/thioanisole/EDT/phenol/water (82.5/5.0/2.5/5.0 v/v/v/v) at room temperature for 4 h. After filtration, the filtrate was concentrated under argon stream and precipitated with diethyl ether. All synthetic peptides were purified by reserve phase chromatography using a semi-preparative HPLC system, RP-HPLC (Shim-pack Prep-ODS; Shimadzu, Kyoto, Japan), and the purity and identity of the peptides were confirmed by MALDI-TOF MS in an Ettan MALDI-TOF/Pro instrument (GE Healthcare, Uppsala, Sweden) and by analytical RP-HPLC using two different solvent systems.

Bothropasin was obtained as described elsewhere [17]. Fibroblast Growth Factor-basic human recombinant protein was from Sigma (St. Louis, MO, USA), Collagen type I from rat-tail was from Roche (Basel, Switzerland), Human plasma Fibronectin was from Gibco (Grand Island, NY, USA), Fibrinogen was from Kabi (Basel, Switzerland), recombinant human VEGF was from Biosource (Camarillo, California, USA). Phospho-p44/42 MAPK (Erk1/2) (Thr202/Tyr204), p44/42 MAPK (Erk1/2) (137F5), Phospho-Akt (Ser473), Akt (#9272) and Beta-Actin antibodies were from Cell Signaling (Danvers, MA, USA). Anti-Integrin alpha2+beta 1 [16B4] ab30483 and Anti-Integrin alpha V beta3 [27.1 (VNR-1)] ab78289 were from Abcam (Cambridge, UK), HRP-conjugated secondary antibodies were from Santa Cruz Biotechnology, the gamma-secretase inhibitor DAPT was from Sigma.

### Fibrinogen cleavage by bothropasin and peptide pool recovery

Incubations were performed in 500 μL H_2_O containing 100μg fibrinogen in the presence (Fgn Bt) or absence (Fgn control) of 2 μg bothropasin for 1h at 37° C. After incubation, 3 volumes (1500 μL) of iced acetone were added to the solutions, which were kept at −20° C overnight for protein precipitation. Tubes were centrifuged at 12000 g at 4° C for 10 minutes, the supernatants were dried in the speed vac and resuspended in PBS (200 μL) for fluorescamine dosage, functional analysis in proliferation and migration assays and HPLC fractioning. Concomitantly, the pellet was recovered and analyzed using a 12.5% SDS polyacrylamide gel carried out according to Laemmli, 1970 [43], for verification of the fibrinogen proteolysis by bothropasin upon separation of the remaining fibrinogen chains.

### HPLC fractioning

The peptide pools obtained in the previous item (Fgn and Fgn Bt) were fractionated by C18 reserve phase chromatography on High-Performance Liquid Chromatography (HPLC). The sample was eluted with a 5–70% buffer B gradient for 80 minutes at 1 mL/min flow, being buffer A 0.1% TFA and buffer B acetonitrile in 0.1% TFA (9:1). After analysis of the chromatographic profile, the subfractions enriched in the Fgn Bt sample were recovered for testing in BrdU incorporation tests allowing the identification of the active subfraction for mass spectrometry analysis.

### Mass spectrometry analysis for peptides identification

The peptide pools were lyophilized, dissolved in 0.1% trifluoroacetic acid, subjected to ZipTipC18 (Millipore Co., Bedford, MA, USA), and spotted onto the sample plate of Ettan Matrix-Assisted Laser-Desorption Ionization - Time of Flight (MALDI-TOF)/Pro mass spectrometer (GE Healthcare, Uppsala, Sweden) mixed with the same volume of a saturated solution of γ-cyano-4-hydroxycinnamic acid in 50% acetonitrile/0.1% trifluoroacetic acid, and analyzed using P_14_ R [(M+H)^+^ 1533.8582] (Sigma) as external calibrates. The peptide pools were also injected into a nanoAcquity UPLC system (Waters) using a 75 μm × 100 mm, nanoAcquity UPLC BEH 130 C18 column (1.7 μm particle size; Waters) coupled to a nano-ESI Q-TOF Ultima API (Waters). The equipment was operated in positive ionization mode using nitrogen as nebulizer gas and 0.1% phosphoric acid as calibrant. Peptides were eluted with a linear gradient of 0–30% B in 45 min (phase A: 0.1% formic acid in water; phase B: 0.1% formic acid in ACN) at 600 nL/min. Capillary and cone voltages were set to 3.5 kV and 100 V, respectively. MS spectra were acquired for 1 s from *m*/*z* of 250–2000 and three of the most intense doubly or triply charged precursor ions were selected for MS/MS analysis. MS/MS spectra obtained from CID with argon were acquired for 2.5 s from *m*/*z* of 50–2000. The collision energy was automatically set according to the *m*/*z* and ion charge varying from 15 to 56 eV. A dynamic peak exclusion was applied to avoid the same *m*/*z* to be selected for the next 45 s [44].

### Cell cultures

Immortalized human umbilical vein endothelial cells (HUVEC-CS - CRL-2873™) and B16-F10 (murine melanoma cells; CRL-6475) were purchased from ATCC (Manassas, VA, USA) and routinely grown in Dulbecco Minimal Essential Medium (DMEM) containing 20% fetal bovine serum (FBS), 1 unit/mL penicillin, 1 μg/mL streptomycin and 5 μg/ml gentamicin (Sigma) and maintained at 37° C in 95% humidified atmosphere, containing 5% CO_2_.

### Cytotoxicity and proliferation assays

To assess the peptides cytotoxicity in HUVEC-CS the MTT (3-[4,5-Dimethylthiazol-2-yl]-2,5-diphenyltetrazolium bromide) assay was performed. The cells were seeded into 96-well microtiter plates at 3 × 10^4^ cells/mL in DMEM containing 20% of FBS in the presence of various concentrations of alpha-C and SCR peptides for 24 h, 48 h, and 72 h. At these time points a quarter volume of MTT solution (2 mg MTT/mL phosphate-buffered saline [PBS]) was added to each well, and each plate was incubated for 4 hours at 37° C resulting in an insoluble purple formazan product. The medium was aspirated and the precipitates dissolved in 100 μL of dimethyl sulfoxide (DMSO) buffered at pH 10.5. The absorbance was then read at 540 nm. In order to assess peptides effect on HUVEC-CS proliferation a colorimetric BrdU incorporation assay (Roche, Mannheim, Germany) was performed. Briefly, the cells were serum starved for 48 h, then subjected to experimental treatments for 12 h, and then BrdU solution (10 μM) was added to the cells for an extra 14 h. Next, cells were fixed and the DNA was denatured in one step by adding fixDenat solution for 30 min. Incorporated BrdU was detected by an anti-BrdU-POD antibody within 60 min. The immune complex was detected by a subsequent substrate reaction and quantified by measuring the absorbance at 450 nm (reference wavelength 550 nm).

### Migration assay

Cells were plated at the density of 3 × 10^4^ cells/mL in DMEM with 20% FBS and allowed to form a confluent monolayer. The cell layer was scratched using a p-200 pipette tip. DMEM containing different concentrations of the peptides alpha-C and scrambled or 20 ng/mL bFGF, 40 ng/mL VEGF, 20% FBS was added and the cells were allowed into the scratched area and were monitored over the course of 72 h. Images were taken using a Nikon Eclipse Ti microscope (Nikon, Melville, NY, USA). Cell motility was quantified by measuring the distance between the migrating cell boundaries, calculating the difference between the mean of five measurements at time zero, and 20 h. Results were normalized by dividing the mean difference in each treatment by the mean difference in the control and expressed as migration index.

### Chemotaxis assay

QCM™ 3 μm Cell Migration Assay – Fibronectin Colorimetric (Millipore) was used according to manufacturer’s instructions. Briefly, 250 μL of serum-free medium in the presence or absence of alpha-C and scramble or 20 ng/mL bFGF, 40 ng/mL VEGF, 20% FBS were placed in the feed tray and 250 mL containing 2.5 × 10^5^ cells were placed in the fibronectin-coated migration chamber. After 12 h of incubation, the non-migrating cells were dislodged from the upper side of the migration chambers and underside migrated cells were stained with cell stain solution. For quantification, stained cells were solubilized in extraction buffer and the OD was measured at 540–570nm. Results are expressed discounting reads of BSA-coated control chambers.

### Cell adhesion assay

Cell adhesion to fibronectin and to Collagen type I were monitored using CytoMatrix™ adhesion strips (Chemicon® international) coated with the substrates or BSA as a negative control. Then, 2.5 × 10^5^ cells were seeded in 100 μL of suspension in the presence or in the absence of the peptides tested for adhesion inhibitory activity at various concentrations. After 1 h of incubation at 37° C, non-adherent cells were removed by PBS washing and stained with 0.2% crystal violet in 10% ethanol. Next, cells were solubilized in a buffer (50% ethanol 0.1 M NaH_2_PO_4_ pH 4.5) for absorbance determination at 540–570 nm.

### Gelatin activity

Six-well culture plates at 80% cell confluence were serum starved for 48 h and treated or not (T0, negative control) for 12 h in the presence of 10 μM alpha-C and 10 μM scrambled peptide or 20 ng/mL bFGF, 40 ng/ml VEGF, 20% FBS. The conditioned medium (CM) was analyzed by gelatin zymography. Briefly, 50μg protein samples of CM were electrophoresed in a 10% SDS-PAGE containing gelatin (2 mg/mL, Sigma). Gels were rinsed in 2.5% Triton X-100 and incubated at 37° C for 20h in 0.15 M NaCl, 10 mM CaCl_2,_ and 50 mM Tris-HCl (pH 7.5) and were stained with 0.05% Coomassie blue and destained in 10% isopropanol and 10% acetic acid.

### Western blot analysis

Cells were grown to 80% confluence in P100 culture plates, serum starved for 48 h and treated for indicated times with 10 μM alpha-C or 20 ng/mL bFGF, or the combination of both agents. Equal amounts of total protein (20 μg) from cell lysates obtained by lysis in a suitable buffer [50 mM Tris-HCl (pH 7.4), 150 mM NaCl_2_, 1 mM EDTA, 0.25% sodium deoxycholate, 1 mM sodium fluoride, 1 mM sodium orthovanate, 0.5 mM PMSF, 10 μg/mL aprotinin, 10 μg/mL leupeptin] were separated by SDS-PAGE and transferred to polyvinylidene difluoride membranes (Bio-Rad, Hercules, CA, USA). After blocking with 5% nonfat dry milk and 0.1% Tween-20 in PBS, membranes were incubated with primary antibodies diluted 1:1000 followed by incubation in HRP-conjugated secondary antibodies (1:5000). Membranes were developed using ECL system (Amersham Bioscience Corp., Piscatway, NJ, USA). Lysates were generated in duplicates in three independent experiments.

### *In vitro* tube formation

Matrigel matrix was added to each well of a chilled 24 well plate and allowed to polymerize for 30 min at 37° C. Next, HUVEC-CS at the density of 10^5^ cells per well in DMEM were plated on the matrigel (Trevigen, Gaithersburg, USA). After a 48 h incubation at 37° C, tube formation was observed using Olympus CK2 light microscope. The total tube length was measured using ImageJ software freely available from the National Institutes of Health at http://rsb.info.nih.gov/ij.

### Immunofluorescent detection

Cell suspensions of 1 × 10^6^ cells/mL were seeded in P100 culture dishes containing previously coated slides (5 μg/mL in PBS fibronectin or collagen type I solutions for 1 h at 37° C and blocked in PBS/BSA 2mg/mL) and serum starved for 48 hours. After 1 hour at 37°, C in a CO_2_ incubator by paraformaldehyde 4% addition for 15 min and cells were permeabilized with TritonX-100 0.1% for 5 min. Primary antibodies were diluted 1:1000 in TBS/BSA 1% and added to slides for 2 h at RT, washed 3 times with TBS under agitation and incubated with Fluorophore-conjugated secondary antibodies (1:500), DAPI (1:5000) for 1 hour at RT protected from light. The slides were washed and mounted using ProLong^®^ Long Gold anti-fade Molecular Probes) and images were acquired using a DXM200 camera coupled to a 2.5× magnification lens and a Nikon E600 microscope with the 100X/1.4plan-apochromatic objective. Four slides per treatment were imaged in two independent experiments.

### FACS analysis

HUVEC-CS (10^6^ cells/well/100 mL) were seeded in 6-well plates and incubated for 12 h, then treated with 10 μM FITC-alpha-C. After treatment, the cells were harvested and washed once with PBS and stained with PE-labeled anti-human antibody or isotype control IgG_2A_ (BD, Biosciences). Samples were stained with anti-alpha_V_beta3 and fixed with 2% paraformaldehyde (PFA)/PBS solution. Fluorescence intensity was measured by flow cytometry (FACScalibur, Becton Dickinson). A total of 10,000 cells/sample were analyzed and the mean fluorescence intensity recorded.

### Chick chorioallantoic membrane assay

Fertilized chicken eggs (*Gallus gallus*, JA57) were incubated at 20° C in an 80% humidified atmosphere. At E2 a window was made in the eggshells and sealed with scotch tape. At E10 alphastatin-C and Scramble peptides (50 μg, 25 μg or 5 μg in 20 μL) were directly applied on the CAM surface, inside a silicon ring (inside diameter = 5 mm, Weber Métaux Plastiques). At E12 images of 3 random fields per egg were acquired and anti-angiogenic activity was evaluated by measuring the number of secondary ectopic sprouts inside the silicone ring by using Image J. Four eggs per treatment were used and three independent experiments were performed.

### Mouse model and tumor growth

B16-F10 cells were grown in RPMI-1640 medium supplemented with 10% FBS, 2 mM l-glutamine, 100 U/mL penicillin and 100 μg/mL streptomycin. After four *in vitro* passages, cells were detached from a flask with 0.1% trypsin and 0.2% EDTA. Then, viable cells were counted based on trypan blue dye exclusion method. B16-F10 cells 5 × 10^4^ cells were suspended in 100 μL of PBS and injected into the dorsal flank of C57BL/6 mice. After 10 days of inoculations, when the tumor implanted in the animals reached a volume of 60 mm^3^, mice (n = 10) were divided into five experimental groups. The treatment was started with the intraperitoneal administration (i.p) of alpha-C at the concentrations of 50 or 100 mg/kg/day and continued for 15 days. The tumor sizes were measured three times a week using a caliper-like instrument during the experiment and converted to tumor weight by the equation: tumor weight = (length2 × width)/2. When the tumor reached 2000 mm^3^, the animal was euthanized by CO_2_ inhalation. All the procedures were conducted in accordance with the guidelines for animal experimentation determined by the Institutional Animal Facility from Butantan Institute (process number 566/09).

### Mouse models and pial collateral remodeling assay

To evaluate whether alpha-C also presents *in vivo* effects in normal vascularization, we used Cx40GFP transgenic mice. This CD1 strain expresses EGFP under control of the arterial promoter Gja5 (connexin 40). Mice were treated by intraperitoneal administration (i.p.) of 100 mg/kg alpha-C, 100 mg/kg DAPT or PBS at postnatal days P2 and P4, and collateral vessel density was assessed at P5 by direct imaging using an Axioskope A1 microscope (Zeiss, Jena, Germany) and a Coolsnap HQ fluorescent camera (Photometrics) connected to MetaMorph (version 7.7.2.0) image analysis software. Alternatively, Dll4^+/-^-deficient CD1 mice carrying a beta-galactosidase for heterozygous identification (described in Duarte et al., 2004) were backcrossed onto Cx40GFP mice and kept on a CD1 strain background, those untreated animals were imaged at P5.

### Statistical analysis

All values were expressed as mean ± SD. Each value is the mean of at least three independent experiments in each group. The differences between the experimental groups were compared by analysis of variance (ANOVA) and Student’s t-tests using GraphPad Prism 4.0. The values were considered statistically significant if the p-value was less than 0.05.
